# Playing Flappy Bird Based on Motion Recognition Using a Transformer Model and LIDAR Sensor

**DOI:** 10.3390/s24061905

**Published:** 2024-03-16

**Authors:** Iveta Dirgová Luptáková, Martin Kubovčík, Jiří Pospíchal

**Affiliations:** Institute of Computer Technologies and Informatics, Faculty of Natural Sciences, University of Ss. Cyril and Methodius, J. Herdu 2, 917 01 Trnava, Slovakia; iveta.dirgova.luptakova@ucm.sk

**Keywords:** reinforcement learning, motion sensors, ray casting, signal processing, time series processing, transformer model, robotics, Flappy Bird game, agent control

## Abstract

A transformer neural network is employed in the present study to predict Q-values in a simulated environment using reinforcement learning techniques. The goal is to teach an agent to navigate and excel in the Flappy Bird game, which became a popular model for control in machine learning approaches. Unlike most top existing approaches that use the game’s rendered image as input, our main contribution lies in using sensory input from LIDAR, which is represented by the ray casting method. Specifically, we focus on understanding the temporal context of measurements from a ray casting perspective and optimizing potentially risky behavior by considering the degree of the approach to objects identified as obstacles. The agent learned to use the measurements from ray casting to avoid collisions with obstacles. Our model substantially outperforms related approaches. Going forward, we aim to apply this approach in real-world scenarios.

## 1. Introduction

The autonomous control of robots using reinforcement learning (RL) has emerged as one of the important topics in machine learning. The extensive use of deep neural network technology has made it the most common choice for creating control systems that rely on information collected from a robot’s operating environment. This paper focuses on processing the collected data within a time framework and using motion information to control the robot’s actions. The architecture used is the transformer model [1], which can efficiently process long time series [2].

The popular computer game Flappy Bird created by Vietnamese programmer Dong Nguyen [3] acts as the simulation environment here. The goal of the player, who controls a simulated robot bird, is to fly continuously forward without a collision. The bird encounters a succession of pairs of pipes obstructing its path, and they are suspended from the top and protrude from the bottom of the game environment. A constant distance is maintained between each pair of pipes, forming a gap through which the bird can fly. The vertical position of this gap is randomly generated, introducing a dynamic element to the game. The ever-changing environment demands that players adapt and quickly react. Gravity pulls the bird downward, whereas the player’s actions push the bird upward. Horizontal velocity remains constant. The game concludes instantly if the bird collides with either a pipe or the ground.

There are several approaches to train players in Flappy Bird. One typical approach is to use the image generated by the game [4], with various adaptations. Another modification involves introducing an extra negative reward when the agent collides with the upper edge of the game screen [5]. Further modifications are based on the creation of three training difficulty levels, easy, medium, and hard [6], which are distinguished by the width of the gap between pipes. The subsequent method involved a computer expert who extracted key information from the pipes and the agent, which is then used to predict actions [7].

In this paper, the player–bird is equipped with a simulated light detection and ranging (LIDAR) sensor represented by the ray casting method to detect pipelines and ground. The player can utilize time-series signal processing to maneuver around pipelines and avoid collisions. The goal is to use motion data to navigate through obstacles, such as pipes and the ground. In this model, a custom-built deep neural network, called here the “motion transformer”, is employed for both time series and ray casting signal processing.

A similar approach to processing temporal components is used by [8,9,10]. However, these papers primarily focus on processing human activity data while also incorporating spatial components. All spatial measurements are interpreted as features. The transformer model in the present study is only looking for time correlations and not for correlations between the rays of the sensor.

The transformer model has already been used for action prediction, where it determines the next action based on the current state, previous actions, and rewards. However, this model specifically uses a causal transformer, limiting information processing only to one direction from the past to the future [11]. Another application utilizes the transformer model in RL as the replacement of convolutional layers for feature extraction. This is the case of the Swin Transformer model used for image processing [12]. It differs from the present paper, which does not incorporate the entire game screen as part of its input. The next application of the transformer model is in the temporal domain, but it only considers using the last timestep for action prediction, while the remaining timesteps are only used in the learning process to compute the model’s error. It also uses a causal transformer [13].

Additional strategies for enhancing time-series prediction involve utilizing the last timestep, averaging features across timesteps, and determining the maximum value across timesteps.

The vision transformer [14] uses the features from the last timestep for action prediction, notably through its use of the class token. In the present paper, the last timestep represents the final state of the game, eliminating the necessity for an additional class token in the time series.

The average of the features across timesteps is used in the paper [15], and their maximum value is reported in [16]. The final alternative involves merging features over time, though this approach may result in a proliferation of inputs to the subsequent layer and contribute to overfitting in the model [17].

In contrast to previous research on Flappy Bird, the present paper aims to use the understanding of the temporal context from ray casting measurements. We employed the transformer model to process historical state measurements, subsequently aggregating these data in a judicious manner to forecast the current action of the agent. The sensor simulated in our study has a more restricted field of view when compared to the methods used in prior research [18]. Therefore, our model is designed to leverage its past knowledge of obstacles within the environment for effective navigation. Unlike neural network models that have been already applied to the Flappy Bird problem, our goal is to devise a method that can effectively condense information transmitted over time. This will allow us to express the qualities of actions for the current state of the agent and its corresponding response. Consequently, our model is designed to predict a categorical distribution of actions based on the current state of the agent, taking into account the agent’s previously evaluated states. This approach allows the model to make informed decisions based on both the current and past states of the agent.

The key contributions of this paper are as follows:

- Improved performance: Our transformer model with a distance sensor significantly outperformed existing methods (an increase of over 50 times in both average and maximum scores). This suggests that real robots equipped with similar sensors can potentially achieve considerably higher accuracy when processing long sequences of sensor data.
- Sensor-focused learning: Unlike previous approaches, our agent solely relies on sensor data (not on the full game image) to learn from past experiences, identify obstacles, and navigate the environment. This suggests that focusing on relevant sensor data can be an efficient strategy for controlling robots.
- Visualizing and tracking the temporal similarity of sensor data: This research introduces a visualization technique to track similarities within sensor data sequences during a transformer’s model training. This technique helps adjust the model to focus on the crucial measurements that impact the game’s strategy and ultimate outcome, effectively discarding non-critical information. This approach was developed to reduce training times and lower memory requirements for the agent.
- Real-world applicability: Our findings have the potential to be applied to real robots operating in hazardous environments (comparable to the Flappy Bird simulation, where the agent can crash). By incorporating a “private zone” concept and deep learning guidance, robots could potentially navigate complex tasks while minimizing collisions and extending their operational lifespan.

This paper is organized as follows. Section 2 provides a review of the core algorithmic and computational approaches employed in this work. These include the dueling network architecture for Q-learning, the motion transformer architecture, the DeepMind Reverb database server used for machine learning, ray casting for obstacle detection, episodic memory incorporated into the transformer’s input, and the private zone concept that aids in obstacle avoidance. Section 3 details the optimization process for the chosen methods and their hyperparameters. This section explores factors such as the number of timesteps used, the feature reduction techniques applied, and the optimal size of the private zone. It concludes with a crash analysis to assess the potential for enhancing the ultimate outcomes. Section 4 discusses future applications of this method and explores promising ways to improve it. Finally, Section 5 summarizes the key findings and conclusions presented throughout the paper.

## 2. Materials and Methods

The transformer model is trained by the dueling deep Q network approach. To achieve effective learning, we need to collect data on various paths explored within the state space and share the updated characteristics of our computational model. This task is facilitated by a specialized DeepMind Reverb database server. The state space only contains measurements from ray casting. The measurements from ray casting therefore warrant a dedicated exposition. Since the transformer is built on episodic memory, its usage in the Flappy Bird problem is independently addressed. Finally, an innovative approach involves the establishment of a private zone surrounding the agent to enhance its ability to maintain a secure distance while navigating obstacles. The introduction of this concept markedly improves performance throughout the learning process. A thorough analysis of these methodologies will be conducted in subsequent sections.

### 2.1. Dueling Deep Q Network

The principle of dueling network architecture is to extract features from the state space that are relevant for value function and advantage function prediction. The value function expresses how advantageous the current state of an agent is for its policy. The agent prioritizes traversing states that possess higher values. This strategy ensures the maximization of the overall value function. In order to make an informed selection among a multitude of potential actions, it is essential to ascertain the benefit associated with each action. This is achieved through the utilization of an advantage function [19]. In the case of a discrete action space, the probabilities of each action need to be expressed in the form of logits, which are predicted by a deep neural network model [20]. Logits represent Q-values, which can be computed according to the following relation [21]:

(1) $$Q\left(s,a\right)=V\left(s\right)+(A(s,a)-\frac{1}{\left|A\right|}\sum_{a^{\prime }}A(s,a^{\prime }))$$

***Q***(***s***, ***a***) expresses the quality function for a given action ***a*** and in a given state ***s***. ***V***(***s***) expresses the value function for a given state ***s***. ***A***(***s***, ***a***) expresses the advantage function for a given action ***a*** in a given state ***s***. The average of the advantage function across actions in a given state ***s*** is subtracted from the ***A***(***s***, ***a***) function. Therefore, the advantage action has a zero mean [22].

The model is trained with the logarithmic hyperbolic cosine (LogCosh) error function, which is less sensitive to outliers than the more conventional mean squared error (MSE) function [23]. The error function of the model is expressed by the following:

(2) $$L\left(\theta \right)=E_{\left(s,a,r,s^{\prime })\sim U(D\right)}[LogCosh(y^{DQN}-Q(s,a;\theta ))]$$

(3) $$y^{DQN}=r+\gamma \underset{a^{\prime }}{\max}Q(s^{\prime },a^{\prime };\theta^{-})$$

U(D) represents the uniform sampling from the replay buffer ***D*** that contains trajectories. Q(s,a;θ) expresses the Q-value predicted by the model. The reward is symbolized by ***r***. $a^{\prime }$ is the next action expressed by the maximum Q-value in the next state $s^{\prime }$. $\theta^{-}$ are parameters of the exponential moving average (EMA) model [24].

### 2.2. Motion Transformer

The motion transformer architecture is based on the encoder block in the transformer model [25]. The purpose of the encoder block is to traverse the input vector across the timeline in both directions. In this way, it is possible to look for relationships in historical data from past to future or from future to past and possibly associate them appropriately with the last timestep. The last timestep represents the source of information in the classical Markov decision process (MDP) [26]. A state vector representing the local memory of the model is fed to the model’s input. The task of the model learning process is then to optimize the global memory (parameters) of the model so that the state space is ideally transformed into an action space. However, the output of the encoder block again represents a sequence; i.e., for each timestep, it predicts a set of extracted features from the input vector. Here, several methods are presented for extracting one particular distribution of the current action ***a_t_***. One possible approach is to only use the extracted features from the last timestep to predict the distribution of actions ***a_t_***, similarly to the class token [27]. The idea is to use the last timestep ***s_t_*** to predict action ***a_t_*** as in classical MDP. If some historical features are needed, they are inserted during the last timestep thanks to the attention mechanism. Another possibility is to use the average or maximum across all timesteps for each extracted feature separately.

**Figure 1:**
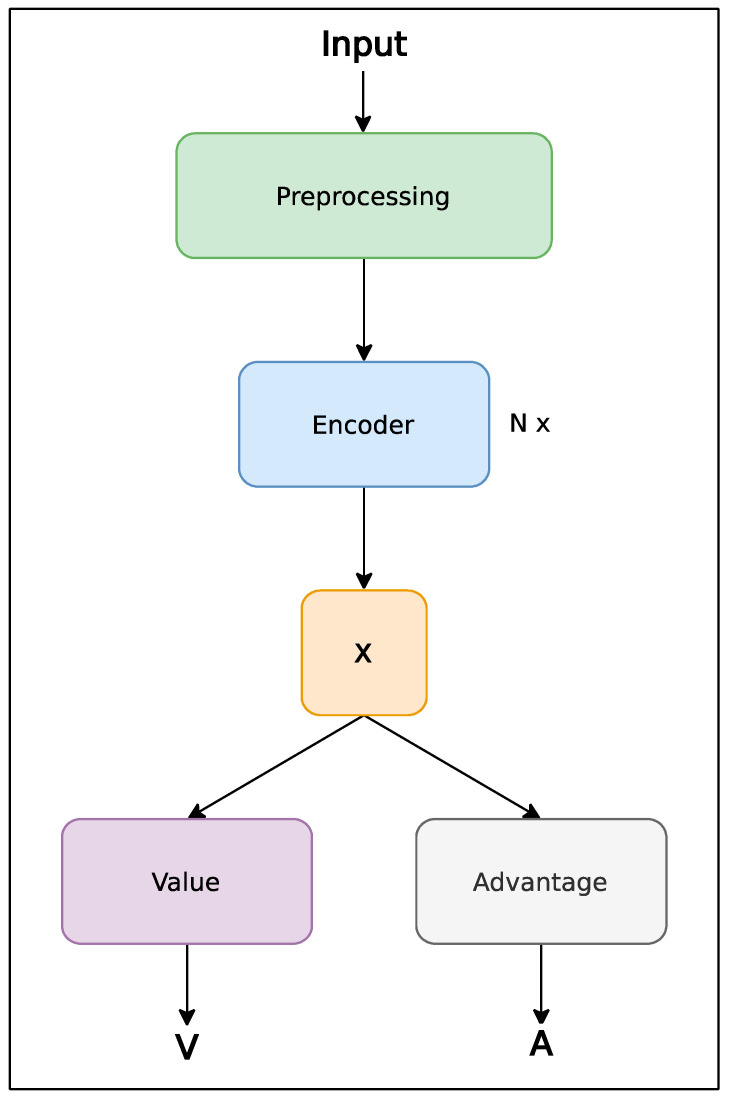
The architecture of the motion transformer model.

Figure 1 shows the architecture of the motion transformer. The architecture consists of a preprocessing layer that adds position information to the input vector within the time series. This is followed by several encoder blocks that extract features along the time axis. The layer labeled X represents the reduction layer of the extracted features across the time series. Its type was varied during experiments. The last layers are value and advantage, representing fully connected output layers. Equation (1) is then applied to the output of the motion transformer.

**Figure 2:**
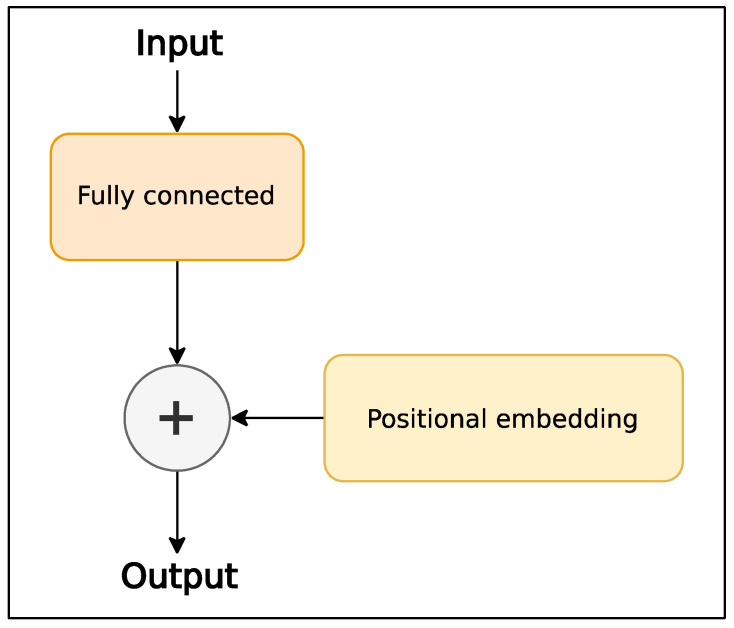
The architecture of the preprocessing layer.

Figure 2 depicts the architecture of the preprocessing layer, which includes a projection layer in the form of a fully connected layer with a linear activation function. Its role is to transform the number of input features into the number of hidden features used in the rest of the model. Subsequently, a positional embedding, which is represented by trainable variables, is summed with the output of a fully connected layer. Thus, in this paper, embeddings for the time series are trained, along with the model [28].

**Figure 3:**
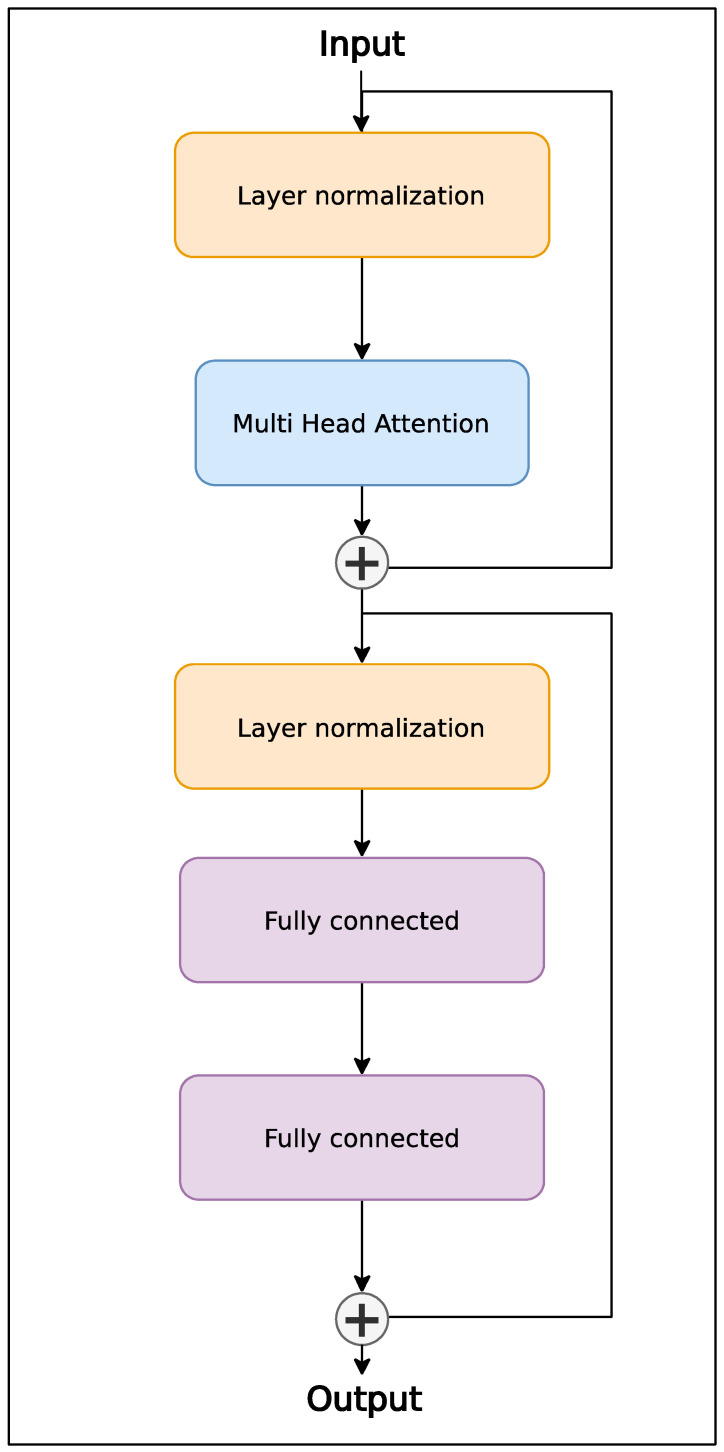
Architecture of encoder layer.

The encoder block architecture is depicted in Figure 3. It consists of a pair of residual [29] sub-blocks. The first is multi-head attention [30], which processes the time series according to the following relations:

(4) $$MultiHead\left(Q,K,V\right)=Concat\left({head}_{1},\ldots ,{head}_{n}\right)W^{O}+b^{O}$$

(5) $${head}_{i}=Attention(QW_{i}^{Q}+b_{i}^{Q},KW_{i}^{K}{+b}_{i}^{K},VW_{i}^{V}+b_{i}^{V})$$

(6) $$Attention\left(Q,K,V\right)=softmax(\frac{QK^{T}}{\sqrt{d_{k}}})V$$

$W^{O}$ represents weights, and $b^{O}$ represents the biases of the linear transformation after merging heads. The process of merging heads comprises concatenating tensors along the head dimension (axis). $W_{i}^{Q}$, $W_{i}^{K}$, and $W_{i}^{V}$ represent weights, and $b_{i}^{Q}$,$b_{i}^{K}$, and $b_{i}^{V}$ represent biases of the linear transformation of the input vector of layer ***Q*** (Query), ***K*** (Key), and ***V*** (Value) into the space handled by the attention function. $d_{k}$ represents the number of dimensions K after the linear projection of the layer input vector.

The second block is a multi-layer perceptron (MLP) [31], and its task is to nonlinearly transform the processed time series. The nonlinear activation function used is Gaussian error linear units (GeLUs) [32], applied after the first fully connected layer. It can be expressed by the following relation:

(7) $$y=GeLU\left(xW_{1}+b_{1}\right)W_{2}+b_{2}$$

$W_{1}$ and $b_{1}$ represent weight and bias parameters for the first fully connected layer to which the nonlinear transformation is subsequently applied. The parameters $W_{2}$ and $b_{2}$ represent the second layer of the block. This layer is responsible for executing a linear transformation on the output derived from the preceding layer. The dimension of this transformed output equals the dimension of the original input vector. Typically, the first layer of the block has 4 times more neurons than the last layer of the block [33].

### 2.3. Database Reverb

The DeepMind Reverb database server is used to effectively manage the collected trajectories and distribute the updated model parameters. This dedicated database server is tailored for RL algorithms where it acts as a replay buffer. Users can control strategies for selecting and removing elements from the database and options for controlling the ratio between sampled and inserted elements. The database server may contain several tables where trajectories or the parameters of the model are stored. An important feature is the compression of the stored data that the database server provides. In the case of overlapped trajectories, it is important to avoid storing duplicate trajectories [34]. The strategy used for sampling trajectories is the uniform sampler, which selects trajectories from the table with equal probability. The strategy for removing trajectories from a table is the first-in-first-out (FIFO) method. The ratio between sampled and inserted items is empirically set to 32 with 10% tolerance.

**Figure 4:**
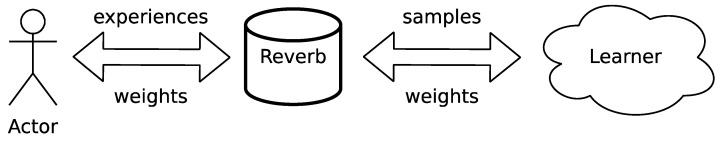
Client–server training.

The client–server architecture used is illustrated in Figure 4. The server represents the database repository where trajectories are stored. The actor represents the client in the form of an agent, which gathers the experience in the form of trajectories through its interactions with the environment and stores the trajectories in the database server. The learner represents a client that retrieves trajectories from the database server and uses them to train an agent model. Following the training process, the agent receives the newly updated model parameters via the database server. A similar principle is used in the Acme framework [35].

### 2.4. LIDAR

**Figure 5:**
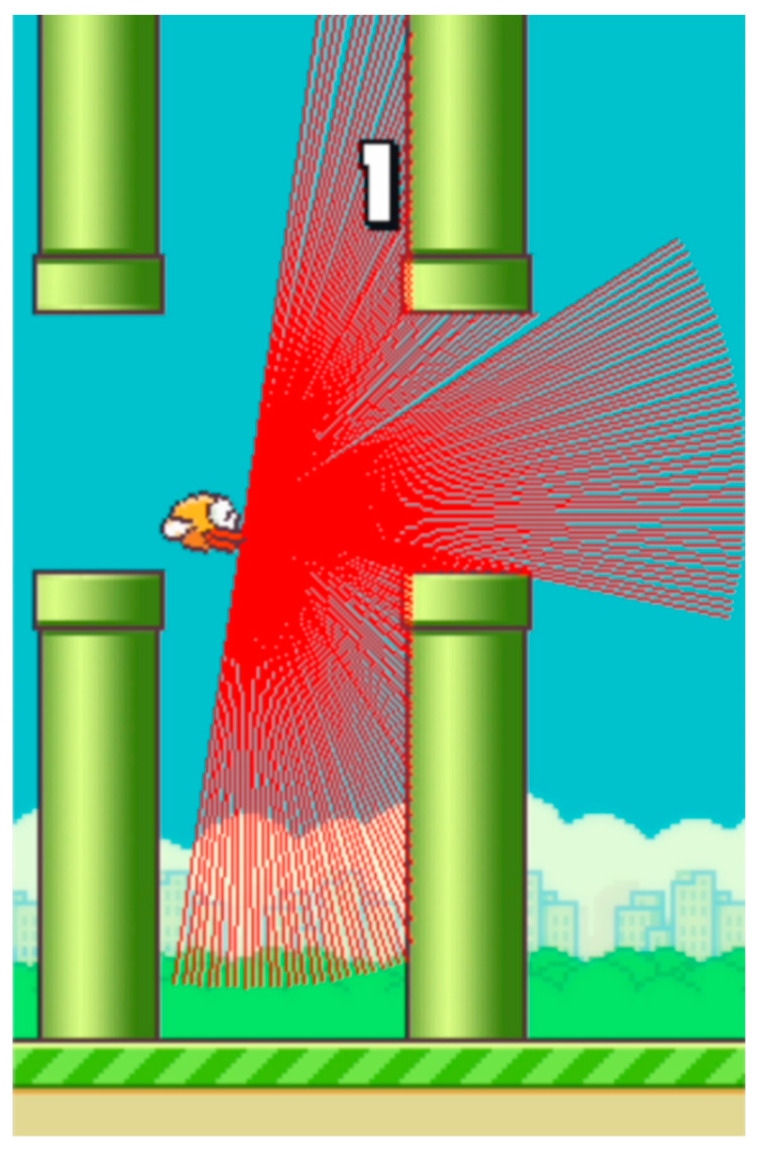
LIDAR sensor represented by ray casting.

The method used to detect nearby objects and track the agent’s movement within the game environment employs a ray casting technique, specifically referred to as LIDAR for simplicity. It consists of 180 rays that are directed from the front of the agent to the right edge of the screen (see Figure 5). The endpoints of rays are determined by the following relations:

(8) $$x=d_{MAX}*\cos\left(\alpha -{player}_{\alpha }-\frac{\pi }{2}\right)+{player}_{x}$$

(9) $$y=d_{MAX}*\sin\left(\alpha -{player}_{\alpha }-\frac{\pi }{2}\right)+{player}_{y}$$

$d_{MAX}$ represents the maximum ray’s length. The angle ***α*** determines the direction of ray radiation, and a ${player}_{\alpha }$ expresses the pitch angle of the player to the plane of the game space. The starting point of the ray is expressed by the coordinates of the front of the agents ${player}_{x}$ and ${player}_{y}$.

When the bird is pushed upward, it rotates towards the sky at a 45-degree angle. In the absence of player input, the bird slowly rotates towards the ground until reaching an angle of −90 degrees and then falls straight down.

The maximum ray length is the distance between the front of the agent and the right edge of the screen. Thus, the perpendicular ray touches the edge of the screen (if there are no obstacles) while other rays at a higher or lower angle usually do not reach the edge of the screen. This behavior mirrors the actual spread of ideal light and the measurements of its reflections from ideally reflective objects at different angles. In contrast, when other methods use an image generated by the game, the system sees it as a whole.

Here, rays emitted through ray casting spread out in a semicircular pattern and can detect obstacles within a limited area ahead of the agent. Moreover, if the bird is not positioned at the correct height and orientation relative to the game environment’s plane, the detection rays do not even register the ground. Consequently, the agent cannot know its altitude throughout each episode. The sensor operates at an angular resolution of 1 degree and has a range limited only by the visible part of the environment ahead of the player. Collision with a ray occurs when the ray hits the surface of a pipe or the ground. The distance to the object is measured as the Euclidean distance between the agent’s front, where the ray originates, and the collision point. However, these values are not statistically optimal for the model’s input; hence, it is convenient to normalize them to the range [0, 1].

(10) $$d_{\alpha }^{norm}=\frac{d_{\alpha }}{d_{MAX}}$$

$d_{\alpha }^{norm}$ represents the normalized distance to the object at an angle ***α***. $d_{\alpha }$ expresses the measured value of the distance to the object. $d_{MAX}$ represents the maximum ray’s length. The $d_{MAX}$ is defined as follows:

(11) $$d_{MAX}=0.8*{Screen}_{w}-{Player}_{w}$$

${Player}_{w}$ represents the width of the agent. The following measurements are given in pixels. The agent has a width of 34 and a height of 24. ${Screen}_{w}$ represents the width of the screen. The screen is the visible part of a game environment that can be seen when the game is rendered. The screen width is 288, and the height is 512.

### 2.5. Episodic Memory

The agent’s state space consists of a fixed-length window of measurements from the timestep history. Therefore, it is necessary to create memory to store these measurements during a game episode. The entire contents of this memory serve as an input vector for the motion transformer. The first-in-first-out (FIFO) data structure ensures the flow of information in one direction, expressing the passage of time in the game environment. As new measurements are acquired during the episode, they replace the oldest ones in the queue. At the beginning of each episode, the queue is initialized with the initial state of the environment. While similarities are observed in the intended result relative to Atari stacking frames at the channel level [36], the present approach introduces a new timestep dimension in the input vector. This enables the model to exploit temporal relationships among measurements. The size of the memory determines how far back the model can effectively analyze measured states in the local history. Insufficient memory capacity can hinder information availability and impede effective action prediction. Conversely, excessively large memory unnecessarily drains computational resources.

**Figure 6:**
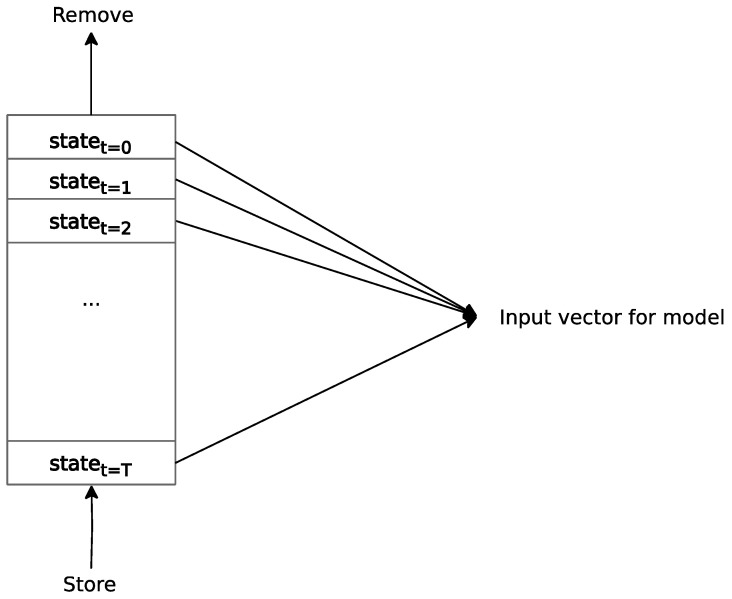
Architecture of episodic memory.

Figure 6 illustrates the principle of applied episodic memory. The new state arrives at the end of the queue from the bottom. The oldest state leaves from the beginning of the queue, i.e., the top part. The input to the motion transformer represents all items that are stored in the queue and are ordered as they come in.

### 2.6. Private Zone around the Agent

In experiments, it was found that the agent tends to take risks and moves too close to the edges of the pipe when passing through the gap between pipes. There is a possibility of penalizing the agent for risky behavior in the policy. Therefore, a penalty for an obstacle approaching inside the agent’s private zone was introduced. With this penalty in place, the agent is motivated to find the optimal solution for the given problem while considering its proximity to recognized obstacles. In a real-world application, object recognition would typically involve a dedicated deep neural network, which aims to distinguish between obstacles and desired objects, such as food or coins, in other gaming scenarios [37].

The agent needs to maintain a safe distance while navigating through obstacles to ensure its policy is not risky. This distance can be experimentally determined by finding the optimal radius for the private zone, which is represented by a circle. The circular model is chosen due to the sensor data being obtained in a circular polar grid format. As the rays are emitted from the agent’s surface, the center of the private zone circle must coincide with the sensor’s center. In the case where the simulation contains obstacles and objects the agent may need to interact with, such as collectibles, it is necessary to define this private zone dynamically. The classification process of obstacles vs. collectibles can be complex and involve sophisticated methods [38,39], and keeping the identified object between consecutive measurements can require specialized methods [40,41]. However, in the present game environment, where only obstacles exist, simple classification suffices.

(12) $$r=\frac{MAX\left({Player}_{w},{Player}_{h}\right)+x}{2}$$

The radius of the private zone circle is defined as ***r***, where ${Player}_{w}$ represents the width of the agent and ${Player}_{h}$ represents the height of the agent. Hyperparameter ***x*** specifies the size of the private zone. Since the rays radiate from ethe dges of the agent and not its center, when x is set to 0, the private zone’s radius is equal to half of the agent’s maximum dimension.

**Figure 7:**
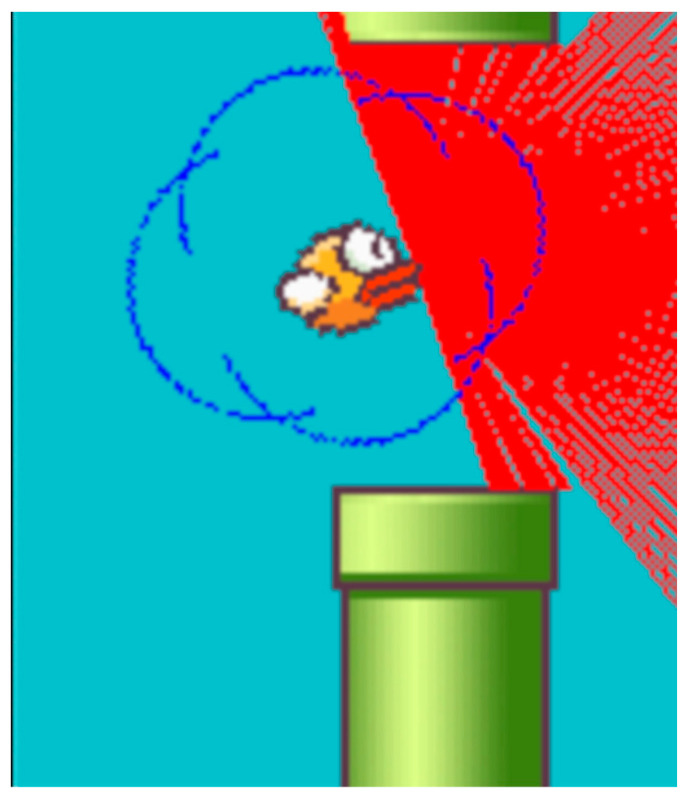
Agent’s private zone.

Figure 7 illustrates the agent’s private zone, where the parameter ***x*** is set to 30. The gap between pipes is fixed at a size of 100 units (i.e., pixels), and the width of each pipe measures 52 units. As can be seen, a high value of ***x*** penalizes the agent if it attempts to navigate through the gap between pipes. Conversely, an ***x*** value that is too low diminishes the existence of a private zone, prompting the agent to take increased risks.

## 3. Results

In order to improve the performance of the deep neural network controlling Flappy Bird’s obstacle avoidance, various techniques required finetuning. This included selecting the right control system architecture and algorithmic techniques, as well as choosing appropriate hyperparameters during implementation. The following section provides detailed explanations of the key aspects of this process.

One aspect involved optimizing the number of timesteps retained in episodic memory. This determined the extent to which the agent would recall the short-term history and utilize it in its action predictions. Additionally, the study focused on refining the architecture of the model. Specifically, it explored whether it was effective to use the last timestep of the output series of actions. Alternatives included the global average or global maximum pooling. These operations involve computing the average or maximum of features across the timestep axis. These methods are commonly employed for reduction tasks, as seen in vision transformers and convolutional neural networks. Lastly, attention was directed towards determining the optimal size of the private zone, with options set to 30, 15, and 0.

**Figure 8:**
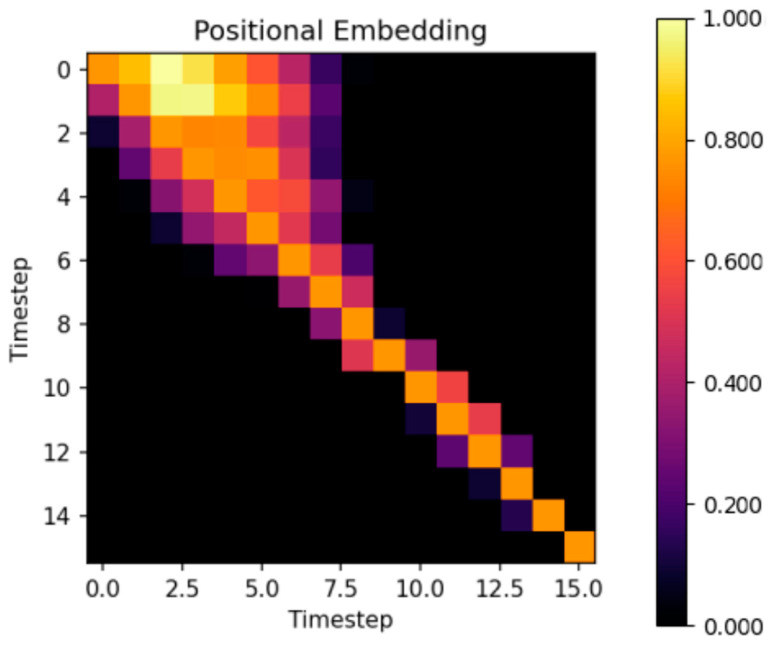
Similarity of timestep embeddings between 16 different timesteps.

The first tested configuration used 16 timesteps as the episodic memory size. Figure 8 shows the cosine similarity between embeddings for different pairs of timesteps. The closest similarities are in the region of the upper-left corner of the heatmap. Therefore, the subsequent experiment aimed to decrease the number of timesteps to diminish the density of similarities among timesteps and refine the optimal number of timesteps. Since there exists similarity among the initial timesteps, it is feasible to restrict their number. A total of 12 timesteps were used, which was anticipated to decrease the density of similarities, particularly in the upper-left corner.

**Figure 9:**
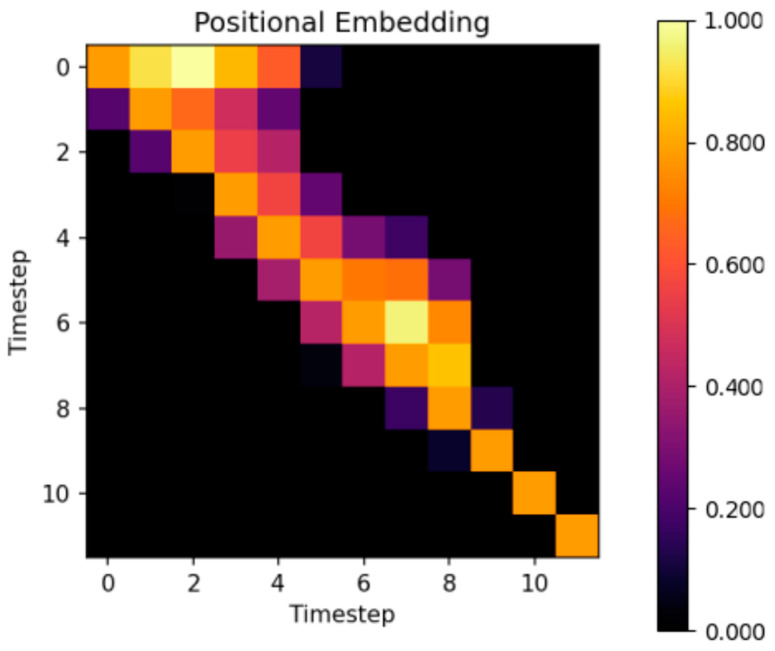
Similarity of timestep embeddings between 12 different timesteps.

The second experiment was to use only 12 timesteps. The cosine similarity between timestep embeddings is depicted in Figure 9. In contrast to using 16 timesteps, the density of timestep embedding similarities in the upper-left corner decreased, but the score of the agent did not significantly deteriorate. Figure 9 is not merely a subset of Figure 8; the difference in the density of similarities is apparent. Additionally, the similarity distribution is not perfectly symmetrical along the diagonal, with past steps showing more resemblance to future steps, especially for distant timeframes. This trend, however, is not observed in recent timesteps. Based on these observations, it could be beneficial in future experiments to explore using fewer past timesteps, as they exhibit similarities to future steps. Timesteps fewer than 12 or higher than 16 were not tested in this study.

In our investigation, we found that as the timestep increases, the similarity of embeddings decreases. This trend is particularly evident in the final timestep, regardless of whether there are 16 or 12 timesteps configured. Typically, the Markov decision process is applied only to the current state ***s_t_***. This implies that the most unique timestep must be the last timestep, which was also supported by measurement with both the 16 and 12 timestep configurations. Specifically, the last timestep exhibits the highest similarity only relative to itself. In this paper, a typical Markov decision process is modified. The historical states and current state are used simultaneously ***s_t-N:t_***, with the exception of the first state ***s_t_*** due to its lack of existing history. Some historical states can probably have similar meanings for the agent. This adaptation draws parallels with word embedding, where words with similar meanings have a higher positive cosine similarity, but on the other hand, words with much different meanings have a small cosine similarity near zero [42].

In the following measurements, the comparison of the last timestep, global average, and global maximum pooling used data collected from 500 episodes. The average and maximum scores across episodes were measured for a deterministic, pre-trained agent. The score represents the number of pipes that the agent successfully passed through.

Table 1 shows the results of comparisons between different reduction techniques. The average of features along the timestep axis is significantly better than other approaches.

Table 2 presents a comparison of the best results achieved in both the highest score and average score in this paper in contrast to other papers. This paper has significantly better scores.

**Figure 10:**
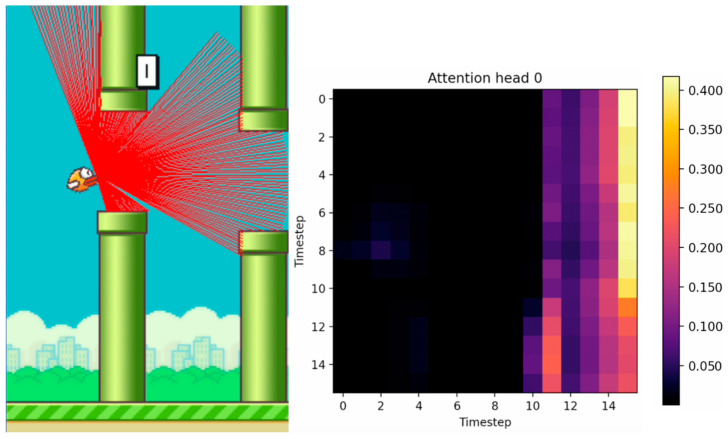
Agent with 16 timesteps entering the gap between the upper and lower pipes. (The brighter the color, the higher the attention value).

**Figure 11:**
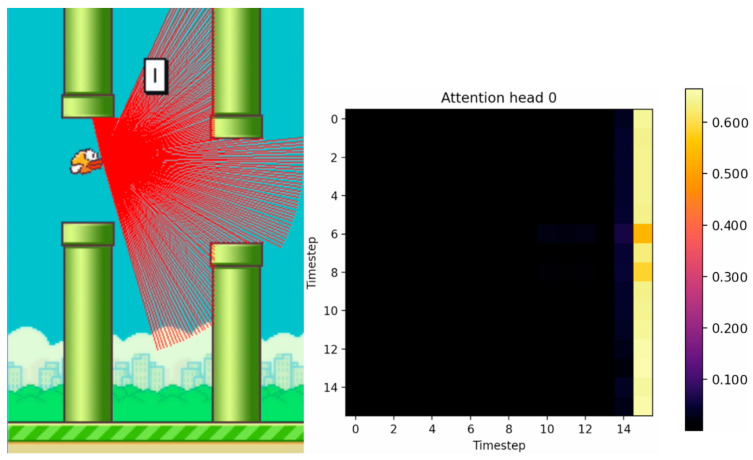
Agent with 16 timesteps in the gap between the upper and lower pipes. (The brighter the color, the higher the attention value).

**Figure 12:**
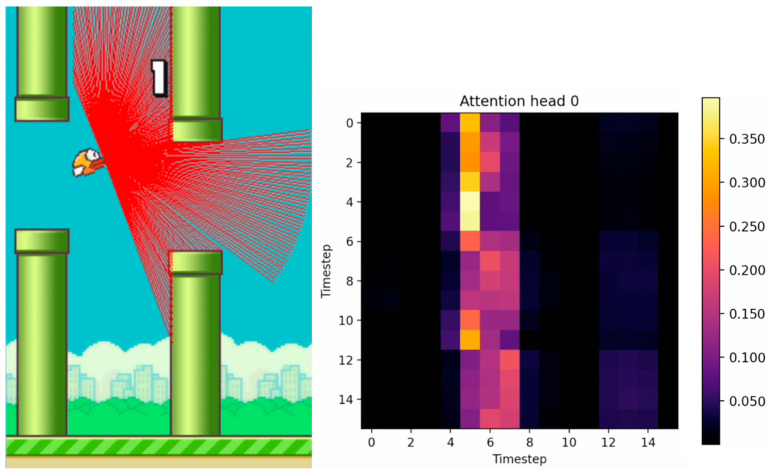
Agent with 16 timesteps passed the gap between the upper and lower pipes. (The brighter the color, the higher the attention value).

Figure 10, Figure 11 and Figure 12 show the tracking of the shifting pipelines along the timeline. The agent uses the history from 16 timesteps. A link to a video showing an animation of the changing attention matrix along with the changing environment is provided in the Supplementary Materials.

From the analysis of the agent’s policy, it is evident that the agent takes risks and approaches the upper or lower pipes while passing through the gap between the pipes. To address this issue, it is necessary to designate a zone for the agent, beyond which, if obstacles are detected, the agent incurs a penalty of −0.5.

Likewise, the same −0.5 penalty stipulated in [5] for the agent reaching the top of the screen is also applied to the obstacles in the agent’s private zone. In this game environment, all objects are regarded as obstacles.

Conversely, if the agent maintains a distance from the obstacles that is above a critical threshold, it is rewarded with a “still alive” reward valued at +0.1, similarly to [44].

**Figure 13:**
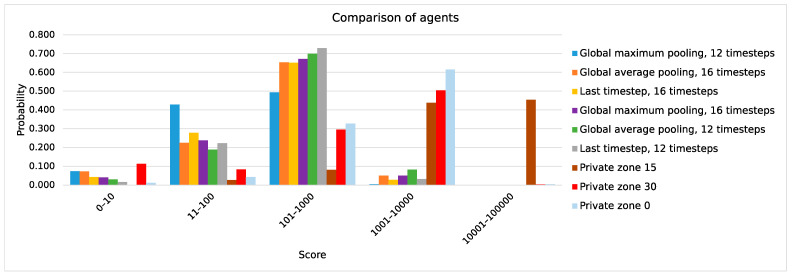
Histogram of score.

Figure 13 shows a histogram of the agent’s score across various feature reduction techniques and private zone sizes. Experiments involving different feature reduction methods did not incorporate a penalty in the reward function for approaching obstacles too closely. Meanwhile, experiments with varying private zone sizes utilized global average pooling with 16 timesteps for feature reduction. Comparing the use of global maximum pooling to global average pooling, it is evident that the agent has a higher likelihood of scoring below 10 when employing the former method. The deep Q network generally overestimates the predicted Q-values [45]. Consequently, employing global maximum pooling may result in an overestimation of Q-values and a more risk-prone policy for the agent.

In the present study, it was observed that when only the last timestep was utilized, akin to the class token in the vision transformer [46], the global average pooling performed similarly to global maximum pooling.

The most stable control of Flappy Bird among the tested options of feature reduction was achieved via the global average pooling reduction method. It provided the highest maximum scores and average scores compared to the other methods of feature reduction. In contrast to global maximum pooling, global average pooling weighs down the activation by combining maximal and non-maximal activations [47]. This behavior leads to a reduction in the overestimation of Q-values predicted by the model and a less risky policy for the agent.

It was observed that using the optimal private zone size resulted in the agent achieving scores that were many times higher. The probability of obtaining a score of less than 100 was extremely low. Furthermore, a high probability of obtaining a score greater than 1000 was observed compared to agents without a private zone.

Table 3 presents a comparison between different private zone sizes. From a selection of several options, results indicate an optimal private zone size of 15. Excessively large values of private zone sizes would also penalize the agent for flying through the pipeline gap until it passes its center, which counts as a high positive reward of +1.0 to the exclusion of the other values of the reward function [48].

**Figure 14:**
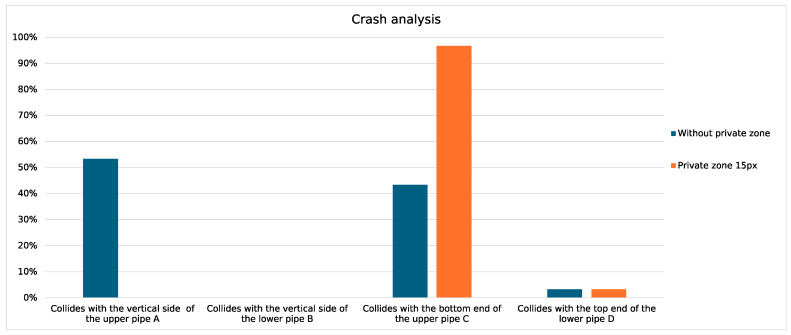
Crash analysis with and without the private zone.

Figure 14 presents the crash analysis for the collisions of the Flappy Bird without the private zone and with the optimal size private zone. While the score with the private zone is better by several orders of magnitude, the crush analysis shows that there still exists room for improvement. A robust solution should have an equal probability of hitting potential obstacles, while the results show that the Flappy Bird tends to crash almost exclusively into the bottom end of the upper pipe. The introduction of the private zone has minimized potential impact points, allowing future focus on suppressing these collisions. One approach to achieving this goal is to design a more robust reward function.

Table 4 displays the hyperparameters used in all the experiments performed. Their values are set based on a combination of recommended settings. The recommended size of the replay buffer and the discount factor are taken from [49]. The multiplier of the MLP block dimension, type of learning rate schedule, and gradient clipping are based on [50]. A private zone with a value of None indicates the absence of a penalty rule for approaching obstacles in the reward function. The other numerical values of the private zone size express the ***x*** of Equation (12).

## 4. Discussion

Utilizing a transformer neural network to control a simulated agent via ray casting as a simple LIDAR sensor has potentially diverse applications across several domains. Remote sensing technology integrated with advanced AI-based control can be beneficial in the following contexts:

In virtual reality and games, avatars or characters can benefit from more natural and responsive interactions.

The method for improving navigation using ray casting in 2D could potentially be expanded to utilize true LIDAR in 3D space. In the future, this could lead to advancements in robotics; autonomous vehicles like self-driving cars, drones, or any kind of mobile robots that require effective navigation; and obstacle avoidance capabilities. In disaster-stricken areas, such robots can aid in search and rescue missions. Enhanced agents can streamline tasks such as inventory management and material handling in warehouses. Additionally, robotic arms could better manipulate objects in dynamic environments.

In each of these contexts, the integration of ray casting and transformer neural network control should enable the agent to make informed decisions based on temporal and spatial information.

When considering the selection of algorithmic procedures and hyperparameters, there exists ample room for exploration and experimentation with various possibilities.

When storing high-dimensional states in episodic memory, it would be more convenient to only extract the significant features for storage. For this purpose, an AutoEncoder-type model could be used to compress the input vector.

Another consideration is the initialization of episodic memory. Currently, it duplicates the initial state, but one alternative includes creating an embedding for the empty state containing episodic memory at each episode’s start. The next option is to dynamically adjust the number of timesteps with respect to the input to the motion transformer, while ensuring the proper assignment of positional embedding for incrementing states from the timesteps.

A promising research direction is exploring the impact of cosine similarity on the optimal number of timesteps. This involves investigating whether the similarity of timestep embeddings can reduce the necessary number of timesteps. Further study is needed to validate the effect of positional embedding in reducing timesteps across various game environments.

In the case of the Flappy Bird game, future research should also try the possibility of adding a weighted reward for keeping a safe distance from the upper pipe more than from other obstacles. This research direction follows from the results of the error analysis. The subject of further research is also to study and rectify failures after the agent has performed a very large number of steps in the environment. Potential improvements might be anticipated in algorithms based on the deep Q Learning principle, such as dueling deep Q learning or double deep Q learning. Numerical instability should also be checked, as well as more advanced Actor–Critic-type models such as A2C or PPO.

Further improvements could be attained by establishing a dynamic private zone around the agent. The private zone could be delineated by deep neural network prediction, whether objects crossing the private zone boundary are obstacles or aids in achieving a specific task. Such a model could directly adjust the complex reward function necessary for task completion without exposing the agent to risky behavior.

## 5. Conclusions

Our study presents novel guidance control using LIDAR sensors represented by the ray casting method for obstacle detection and agent navigation within obstacle-filled environments. The designed motion transformer model effectively grasped the temporal dynamics between sensor readings. The findings demonstrate the model’s ability to adaptively respond to the agent’s movement among pipelines, as reflected in the attention matrix. The model’s attention mechanism prioritizes past or present sensor data, or a combination thereof, based on the spatial distribution of pipelines in the surroundings. Additionally, the results show that employing average reduction techniques helps mitigate the risk of overestimating Q values. Furthermore, the incorporation of a private zone for the agent contributes to the formulation of a less risky navigation policy.

In this paper, the average score (the number of passes through pipeline gaps) obtained by the agent without a private zone is 182 percent better compared to the best results obtained by the competitors. The highest score achieved by the agent without a private zone compared to the best competitors’ obtained results is 199 percent better. The agent with a private zone of 15 pixels achieved an average score that was 6286 percent better than the best competitors’ average agent score and a maximum score that was 5014 percent better than the competition’s best results in terms of maximum agent score.

## Figures and Tables

**Table 1:** Results of tested reduction methods.

Architecture	Timesteps	Highest Score	Average Score
Global average pooling	16	**2970**	324.198
Last timestep	16	2809	286.394
Global maximum pooling	16	1948	329.194
Global average pooling	12	2348	**380.284**
Last timestep	12	1922	335.114
Global maximum pooling	12	1128	152.858

The highest score is emphasized in boldface font.

**Table 2:** Caption.

Paper	Highest Score	Average Score
[43]	15	3.300
[4]	80	16.400
[6]	215	82.200
[5]	-	102.170
[7]	1491	209.298
This paper without a private zone	**2970**	**380.284**
This paper with a private zone	**74,755**	**13,156.590**

The score obtained in this paper is highlighted in boldface font.

**Table 3:** Comparison of scores with different sizes of private zones.

Private Zone	Highest Score	Average Score
None	2970	380.284
0	10,250	2138.858
**15**	**74,755**	**13,156.590**
30	11,383	1645.654

**Table 4:** Hyperparameters of the agent and learner model.

Hyperparameter	Description	Value
port	Database server port	8000
max_replay_size	Maximum database memory	1,000,000
samples_per_insert	Samples per insert ratio for reverb	32
temp_init	Initial Boltzmann temperature for exploration	0.500
temp_min	Minimal Boltzmann temperature	0.010
temp_decay	Decay of Boltzmann temperature	0.999999
warmup_steps	Warmup steps for learning rate cosine scheduler	1000
train_steps	Training steps	1,000,000
batch_size	Batch size	256
gamma	Discount factor	0.990
tau	Tau factor (for EMA model)	0.005
num_layers	Num. of encoder blocks	2
embed_dim	Embedding dimension	128
ff_mult	Multiplier of MLP block dimension	4
num_heads	Num. of attention heads	6
learning_rate	Learning rate	3 × 10^−4^
global_clipnorm	Globally normalized clipping of gradient	1
weight_decay	Weight decay for AdamW optimizer	1 × 10^−4^
frame_stack	Size of short-term (episodic) memory	16 or 12
player_private_zone	Size of agent’s private zone	None, 0, 15 or 30

## Data Availability

Data are contained within the article.

## Supplementary Materials

The following supporting information can be downloaded at the following: interactive charts: https://wandb.ai/markub/rl-toolkit/groups/FlappyBird-v0 (accessed on 16 October 2023); source codes: https://github.com/markub3327/rl-toolkit (accessed on 16 October 2023) and https://github.com/markub3327/flappy-bird-gymnasium (accessed on 16 October 2023); YouTube video: https://youtu.be/aZQxuDCyHoI (accessed on 22 December 2023).
